# Bifurcation study of a neural field competition model with an application to perceptual switching in motion integration

**DOI:** 10.1007/s10827-013-0465-5

**Published:** 2013-09-07

**Authors:** J. Rankin, A. I. Meso, G. S. Masson, O. Faugeras, P. Kornprobst

**Affiliations:** 1 Neuromathcomp Team, Inria Sophia Antipolis, 2004 Route des Lucioles-BP 93, Alpes-Maritimes, 06902 France; 2 Institut de Neurosciences de la Timone, CNRSet Aix-Marseille Université, Campus Santé Timone, 27 Bd Jean Moulin, Marseille, 13385 France

**Keywords:** Multistability, Competition, Perception, Neural fields, Bifurcation, Motion

## Abstract

Perceptual multistability is a phenomenon in which alternate interpretations of a fixed stimulus are perceived intermittently. Although correlates between activity in specific cortical areas and perception have been found, the complex patterns of activity and the underlying mechanisms that gate multistable perception are little understood. Here, we present a neural field competition model in which competing states are represented in a continuous feature space. Bifurcation analysis is used to describe the different types of complex spatio-temporal dynamics produced by the model in terms of several parameters and for different inputs. The dynamics of the model was then compared to human perception investigated psychophysically during long presentations of an ambiguous, multistable motion pattern known as the barberpole illusion. In order to do this, the model is operated in a parameter range where known physiological response properties are reproduced whilst also working close to bifurcation. The model accounts for characteristic behaviour from the psychophysical experiments in terms of the type of switching observed and changes in the rate of switching with respect to contrast. In this way, the modelling study sheds light on the underlying mechanisms that drive perceptual switching in different contrast regimes. The general approach presented is applicable to a broad range of perceptual competition problems in which spatial interactions play a role.

## Introduction

Perception can evolve dynamically for fixed sensory inputs and so-called multistable stimuli have been the attention of much recent experimental and computational investigation. The focus of many modelling studies has been to reproduce the switching behaviour observed in psychophysical experiment and provide insight into the underlying mechanisms (Laing and Chow 2002; Freeman 2005; Kim et al. 2006; Shpiro et al. 2007; Moreno-Bote et al. 2007; Borisyuk et al. 2009; Ashwin and Lavric 2010). Bifurcation analysis (Strogatz 1994; Kuznetsov 1998) and numerical continuation (Krauskopf et al. 2007) are powerful tools from the study of dynamical systems that have already proved effective in analysing rate models where the competing perceptual states are represented by discrete neural masses (Shpiro et al. 2007; Curtu et al. 2008; Theodoni et al. 2011b). Two commonly proposed mechanisms that drive the switching behaviour in these models are adaptation and noise and a strong argument is made in Shpiro et al. (2009) that a balance accounts best for experimental findings across different model architectures and different adaptation mechanisms. Existing studies using discrete neural masses have shown that switching in rivalry experiments can be described by a relatively simple dynamical system. However, the problem of multistable motion integration is different because the perceived direction of motion is represented on a continuous scale. We therefore asked the following questions. Can a minimal model with a continuous feature space describe switching behaviour in motion integration? Do qualitative changes in the dynamics predicted with bifurcation analysis correspond to changes in the mechanisms driving the switches?

Here, we will take advantage of the neural fields formalism (Amari 1971; Wilson and Cowan 1972, 1973) in order to study neural competition in a model with a continuous feature space where adaptation and noise are implemented as mechanisms that can drive activity switches. The model describes the mean firing rate of a population of feature selective neurons. Deterministic versions of this feature-only model with spike frequency adaptation have been studied previously without input (Curtu and Ermentrout 2004) and with a unimodal input (Hansel and Sompolinsky 1998; Folias 2011). A key difference with existing rivalry models is that the competing percepts form tuned responses in a continuous feature space instead of being represented by discrete populations as in, for example, Shpiro et al. (2007), and Theodoni et al. (2011b). The more general model we use allows for perceptual transitions to occur in a smooth way as opposed to discrete switches between two isolated percepts. Starting from the results presented in Curtu and Ermentrout (2004), we will first introduce a simple (unimodal) input and investigate how the various types of solutions from the no-input case are modified. With the application of numerical bifurcation methods we find that although the boundaries between parameter regions featuring different types of responses are gradually distorted with increasing input strength, much of the global structure is preserved. This allows for all possible types of behaviour, and parameter regions for which it can occur, to be comprehensively described across a wide range of model parameters controlling input gain, adaptation gain and the shape of the firing rate function. For a simple input we are able to match the models output to known response properties from the literature before considering the introduction of a complex (multimodal) input that gives rise to multistable behaviour.

In this paper we are interested in moving, ambiguous visual stimuli for which two or more distinct interpretations are possible, but where only one of these interpretations, or percepts, can be held at a time. Not only can the initial percept be different from one short presentation to the next, but for extended presentations, the percept can change, or switch, dynamically. This phenomenon of multistability has been observed and investigated with a number of different experimental paradigms, e.g. binocular rivalry experiments (Levelt 1968; Blake 1989, 2001), apparent motion (Ramachandran and Anstis 1983), motion plaids that are bistable (Hupé and Rubin 2003) or tristable (Hupé and Pressnitzer 2012) and the multistable barberpole illusion (Castet et al. 1999; Fisher and Zanker 2001; Meso et al. 2012b). During extended presentations of these stimuli, the dominant percept switches randomly and the dominance durations between switches have been shown to fit certain distributions dependent on the experimental paradigm (Levelt 1968; Leopold and Logothetis 1996; Logothetis et al. 1996; Lehky 1995; Zhou et al. 2004; Rubin and Hupé 2005).

Here, we will study the temporal dynamics of perception for the so-called multistable barberpole illusion, which has been investigated in complementary psychophysical experiments (Meso et al. 2012b). Some of these results will be presented alongside the modelling work. We will demonstrate how the general neural fields model can reproduce the main dynamical characteristics of the perceptual switches observed in the experiments. We use the mean switching rates reported in the experiments to constrain model parameters and propose specific mechanisms that can account for the behaviour in different contrast regimes. Importantly, we will show that the two contrast regimes identified experimentally, one in which the rate increases with contrast, the other in which the rate decreases with contrast, are linked to specific mechanisms with the model. Although a combination of noise and adaptation drive the switching, the dominant mechanism changes with contrast. Furthermore, we are able to quantify this in an experimentally testable way: the distribution of dominance durations fit different statistical distributions in each contrast regime.

In Section 2 section we give a mathematical description of the model before presenting general results that map the model’s possible behaviours across parameter space in Section 3 and then applying the model to the study of multistable perception in Sections 4 and 5.

## Competition model with continuous feature space

### The neural field framework

The neural field equations provide an established framework for studying the dynamics of cortical activity, represented as an average membrane potential or mean firing rate, over a spatially continuous domain. Since the seminal work by Amari (1971), Wilson and Cowan (1972) and Wilson and Cowan (1973) a broad range of mathematical tools have been developed for their study, see reviews by Ermentrout (1998), Coombes (2005) and Bressloff (2012) along with Ermentrout and Terman (2010, Chapter 11) for a derivation of the equations. The equations describe the dynamical evolution of activity of one or more connected populations of neurons, each defined in terms of a spatial domain that can represent either physical space (on the cortex), an abstracted feature space (orientation, direction of motion, texture preference, etc.), or some combination of the two. This framework has proved especially useful in the study of neuro-biological phenomena characterised by complex spatio-temporal patterns of neuronal activity, such as, orientation tuning in the primary visual cortex V1 (Ben-Yishai et al. 1995; Somers et al. 1995; Hansel and Sompolinsky 1998; Veltz 2011), binocular rivalry (Kilpatrick and Bressloff 2010; Bressloff and Webber 2011), and motion integration (Giese 1998; Deco and Roland 2010).

### Model equations

In this section we describe a general neural competition model that switches between selected states for an ambiguous input. The model describes the time-evolution of a neuronal population defined across a continuous feature space in which a selected state corresponds to a tuning curve. A spatial connectivity is chosen that produces mutual inhibition between competing tuned responses so that a winner-takes-all mechanism leads to a definitive tuned response at any given time instant. Over time, shifts between tuned responses are driven by a combination of adaptation and noise.

We will consider a single neuronal population *p*(*v*, *t*), defined across the continuous, periodic feature space *v* ∈ [−*π*, *π*), whose evolution depends on time *t*. The variable *p*(*v*, *t*) takes values in [0, 1] representing activity as a proportion of a maximal firing rate normalised to 1. We also define secondary adaptation *α*(*v*, *t*) and stochastic *X*(*v*, *t*) variables. The time evolution of *p*(*v*, *t*) is described by the following coupled system of integro-differential equations:

1 $$\begin{array}{*{20}l} \hfill\displaystyle\tau_{p}\frac{\mathrm{d}}{\mathrm{d} t}\,p(v,t) &=& -p(v,t)+S\big(\lambda[J(v)* p(v,t)-k_{\alpha}\alpha(v,t) \\ &+& k_{X} X(v,t)+k_{I}I(v)-T]\big), \end{array} $$

2 $$ \hfill\displaystyle\tau_{\alpha}\frac{\mathrm{d}}{\mathrm{d} t}\,\alpha(v,t) = -\alpha(v,t) + p(v,t). $$

**Fig. 1:**
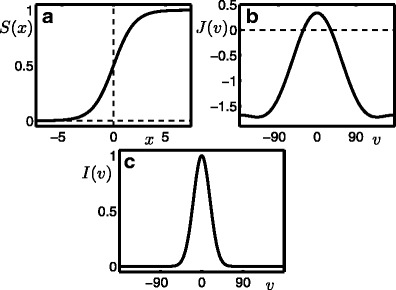
Model features and stimulus. **a** The smooth (infinitely differentiable) sigmoidal firing rate function *S*(*x*). **b** The convolutional kernel *J* is a three-mode approximation of a Mexican hat connectivity. **c** The simple stimulus is a Gaussian bump centred at *v* = 0°

The principal Eq. (1) has time constant *τ*_*p*_ and has a standard decay term − *p*. A smooth, nonlinear sigmoidal firing rate function

3 $$ S(x)=\frac{1}{1+\exp(-x)} $$

is used as plotted in Fig. 1a. The slope and threshold of the firing rate function are controlled by the parameters *λ* and *T*, respectively. The firing rate function processes lateral connections described by *J* and inputs from adaptation *α*, additive noise *X* and a time independent input *I*; the respective input gain parameters are *k*_*α*_, *k*_*X*_ and *k*_*I*_. The connectivity in the feature space *v* is represented by a convolutional operator *J* that approximates a Mexican hat connectivity (local excitation, lateral inhibition). As in Curtu and Ermentrout (2004), we use a 3-mode expansion and *J* takes the form

4 $$ J(v)=J_{0}+2J_{1}\cos(v)+2J_{2}\cos(2v), $$

see Fig. 1b. The adaptation dynamic in Eq. (2) describes linear spike frequency adaptation that evolves on a slow time scale *τ*_*α*_. The additive noise *X*(*v*, *t*) is an Ornstein-Uhlenbeck process that evolves on the same slow timescale *τ*_*α*_ as the adaptation, has mean 〈*X*(*v*, *t*)〉 = 0, variance Var(*X*) = 1 and no feature correlation; see Appendix A for further details. The input *I* depends only on the feature *v* and the so-called simple input studied in Section 3 is shown in Fig. 1c.

### Parameter values, initial conditions and numerical computations

The parameter values used in the numerical computations in each section of the paper are given in Table 1. For the model simulations without noise (*k*_*X*_ = 0), as presented in Sections 3 and 4, we solve the system described by Eqs. (1–2) using the ODE23t solver in Matlab with default settings except the relative tolerance, which was set to 10^−6^. A 200-point discretisation of *v* was used that satisfies error bounds for the computation of the integral term *J* ∗ *p* with a standard trapezoid method. For the simulations with noise ( *k*_*X*_ = 0.0025), as presented in Section 5, the same discretisation is used with a standard Euler-Maruyama method and a fixed timestep of 0.5ms. In all simulations the initial conditions are set to a low level of activity *p*_0_(*v*) = 0.1 (10% of the maximal firing rate) with a small randomised perturbation. Initial conditions are given by

$$ p(v,0)=p_{0}(v),\quad \alpha(v,0)=0, \quad X(v,0)=0. $$

**Table 1:** Parameter values used in the numerical studies

Description	Parameter	Value	Section(s)
Zero-order coefficient of J	*J*_0_	-1	Fixed
First-order coefficient of J	*J*_1_	1/2	Fixed
Second-order coefficient of J	*J*_2_	1/6	Fixed
Sigmoid threshold	*T*	-0.01	Fixed
Firing rate stiffness	*λ*	Free in [12,26]	Sections 3 and 4
…as function of contrast *c* ∈ [0,1]	*λ*(*c*)	[13,25]	Section 5
Adaptation strength	*k*_*α*_	Free in [0,0.07]	Sections 3 and 4
	*k*_*α*_	0.01	Section 5
Input gain	*k*_*I*_	0 or 0.001	Section 3
	*k*_*I*_	0.01	Sections 4 and 5
Noise strength	*k*_*X*_	0	Sections 3 and 4
	*k*_*X*_	0.0025	Section 5
Population time constant	*τ*_*p*_	1ms	Fixed
Adaptation timescale	*τ*_*α*_	100ms	Sections 3 and 4.3
Adaptation and noise timescale	*τ*_*α*_	16.5s	Sections 4.4 and 5

In order to carry out a bifurcation analysis of the system (1)–(2) we use a numerical continuation package AUTO (Doedel et al. 1997) that allows us to compute branches of steady state and oscillatory solutions, and to detect and track bifurcations of these solutions. These computations are carried out in the absence of noise (*k*_*X*_ = 0) and in this case we can take advantage of the 3-mode approximation of *J* in Eq. (4) by expressing *p*(*v*, *t*) and *α*(*v*, *t*) in terms of the same modes plus some orthogonal components $\widehat {p}_{\perp }$ and $\widehat {\alpha }_{\perp }$:

5 $$\begin{array}{*{20}l} p(v,t) &=& \widehat{p}_{0}(t)+\widehat{p}_{1}(t)\cos(v)+\widehat{p}_{2}(t)\sin(v)\\ &&+ \widehat{p}_{3}(t)\cos(2v)+\widehat{p}_{4}(t)\sin(2v)+\widehat{p}_{\perp}, \end{array} $$

6 $$\begin{array}{*{20}l} \alpha(v,t) &=& \widehat{\alpha}_{0}(t)+\widehat{\alpha}_{1}(t)\cos(v)+\widehat{\alpha}_{2}(t)\sin(v)\\ &&+\widehat{\alpha}_{3}(t)\cos(2v)+\widehat{\alpha}_{4}(t)\sin(2v)+\widehat{\alpha}_{\perp}. \end{array} $$

In Veltz and Faugeras (2010) it was proved that as *t* → ∞ the orthogonal components decay to 0. Therefore, we can study steady-state and oscillatory solutions to (1)−(2) by solving a set of 10 ordinary differential equations in $\widehat {p}_{i}$ and $\widehat {\alpha }_{i}, i=0\dotso 4$. The integral term *J* ∗ *p* was computed with the same 200-point trapezoid integration scheme used for in the ODE solver. Periodic orbits were typically computed with 150 mesh points (constants NTST = 50 and NCOL = 3) in AUTO. The reduced description is used only for the computation of the bifurcation diagrams.

## General study of competition model

### Bifurcation analysis and numerical continuation

Bifurcation analysis and its computational counterpart numerical continuation are crucial tools in the study of the neural field equations and dynamical systems in general. When varying a model parameter, a bifurcation is a special point at which there is a qualitative change to the types of response produced by the model. Numerical continuation allows one to locate, classify and track bifurcation points and, in this way to map out the exact boundaries between regions in parameter space with qualitatively different behaviour. This kind of information forms a basis for tuning a model’s parameters; indeed, it is possible to ensure that parameter regions in which a desired behaviour is present are not isolated and ensure robustness with respect to small changes in the model set up. Numerical continuation has been used in general studies of the neural field equations (Veltz and Faugeras 2010; Veltz 2011), to investigate localised states (Laing and Troy 2003; Faye et al. 2013) and in a previous study of the short-term dynamics of the stimulus considered in this article (Rankin et al. 2013). One key advantage of using bifurcation and continuation techniques is that they allow for a model to be brought into an operating regime, close to bifurcation, where the model is most sensitive to changes in its input and where the combination of mechanisms involved in performing complex computations can be revealed. This general philosophy has been used to great effect in studies of orientation tuning in V1 (Veltz 2011, Chapter 9), simplified rate models of neural competition (Shpiro et al. 2007; Theodoni et al. 2011b) and studies of decision making (Theodoni et al. 2011a).

### Bifurcations relevant to this study

At a bifurcation point there is qualitative change to the types of response produced by the model. The types of response can differ in terms of 1) spatial properties, such as being tuned or untuned, 2) temporal properties, such as being steady or oscillatory and 3) stability, where stable implies responses that persist in time and unstable implies responses that are transient. Each type of response is associated with a solution of the underlying equations and the organisation of these solutions in state/feature space governs the dynamical behaviour. The main types of bifurcation that we encounter in this study are the pitchfork bifurcation and the Hopf bifurcation; these so-called codimension-one bifurcations occur at a given point as one parameter is varied. In this model the pitchfork is associated with a transition from a homogeneous state to a tuned state. Hopf bifurcations are associated with transitions from steady to oscillatory responses that can either travel in the feature space (travelling waves), or that remain static but oscillate in amplitude (standing waves). In the parameter plane (as two parameters are varied) these codimension-one bifurcations lie on curves. At points where these curves meet or intersect we encounter codimension-two bifurcations that act as organising centres close to which several solution types can be encountered. Due to the presence of translational and reflectional symmetry properties in the underlying equations when there is no input, the bifurcating solutions encountered have the same symmetry properties (Haragus and Iooss 2010). We will give an account of how these symmetry properties break down with the introduction of an input.

In Curtu and Ermentrout (2004) it was shown that in the absence of input (*k*_*I*_ = 0) the model possesses O(2)-symmetry and as a consequence several types of steady solutions and oscillatory patterns exist in different parameter regimes local to the codimension-two Bogdanov-Takens (BT) point (Dangelmayr and Knobloch 1987). As we will see, this BT point acts as an important organising centre in parameter space. First, in Section 3.3, we will give a summary of what is already known from Curtu and Ermentrout (2004) in terms of solution branches and bifurcation curves that are relevant to this study (the account will not be exhaustive, omitting several bifurcation curves involving exclusively unstable solutions). In Section 3.4, we will introduce an input and describe how the solutions existing in different parameter regions change and how the boundaries of these regions shift in parameter space. Although the system (1)−(2) has previously been studied with a unimodal input in (Hansel and Sompolinsky 1998) and more recently in (Folias 2011), the results we present allow us to build a more complete picture of the model’s behaviour in terms of three parameters relevant to our study, the input gain *k*_*I*_, the adaptation strength *k*_*α*_ and the sigmoidal slope *λ*.

### No input (*k*_*I*_ = 0)

**Fig. 2:**
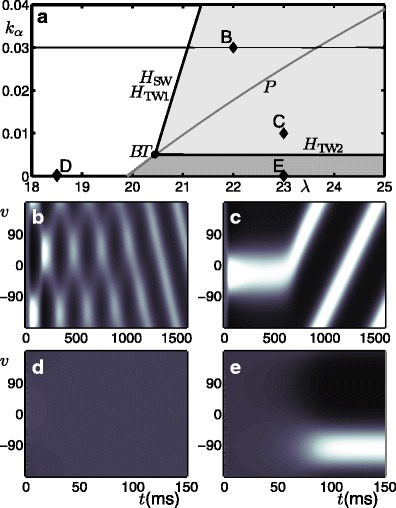
Bifurcation diagram for the no-input case; summary of results from Curtu and Ermentrout (2004). **a** Bifurcation curves plotted in the (*λ*, *k*_*α*_)-parameter plane demarcate regions with qualitatively different dynamics. The Hopf-type curves are *H*_SW_, *H*_TW1_ (coinciding) and *H*_TW2_, a pitchfork curve is *P* and these curves meet at the Bogdanov-Takens point *BT*. Panels **b**–**e** show the activity *p*(*v*, *t*) indicated by intensity for model simulations at parameter values from the corresponding points B–E in panel **a**

Figure 2 shows the different types of dynamical behaviour produced in different regions of the (*λ*, *k*_*α*_)-parameter plane as demarcated by bifurcation curves. The model simulations shown in panels b–e were performed at the corresponding points B–E in panel a. In each case the simulation time was chosen such that the model reaches its stable behaviour during the simulation; the stable behaviour is either a steady state or an oscillatory state. In the white region the model produces an homogeneous (untuned) steady-state response at a low level of activity as shown in panel d. In the dark-grey region the model produces a steady-state response tuned to an arbitrary direction as shown in panel e. The boundary between the white and dark-grey regions is a pitchfork curve *P*; as *λ* is increased and the pitchfork bifurcation is encountered, the homogeneous steady-state becomes unstable and a ring of tuning curves forms the stable behaviour. In the light-grey region the stable behaviour is a travelling-wave solution with an arbitrary direction in *v*; the transient behaviour observed before reaching this stable state changes dependent on the chosen parameter values; see panels b and c. The boundary between the white and the light-grey regions is the coinciding Hopf-type curves *H*_SW_ an *H*_TW1_. As *λ* is increased and the two coinciding bifurcation points are encountered the homogeneous steady states lose stability and two new branches bifurcate simultaneously: an unstable branch of standing wave solutions and a stable branch of travelling wave solutions; this is shown explicitly in Appendix B. In panel b, close to these curves, the unstable standing wave solution is seen as a transient behaviour before eventual convergence to the stable travelling wave solution. The boundary between the dark-grey and light-grey region is *H*_TW2_and as *k*_*α*_is increased and the bifurcation is encountered the stable tuned response becomes spatially unstable and starts to travel in an arbitrary direction. In panel c the unstable tuned response is seen as a transient behaviour before starting to travel.

### Simple input with *k*_*I*_ = 0.001

**Fig. 3:**
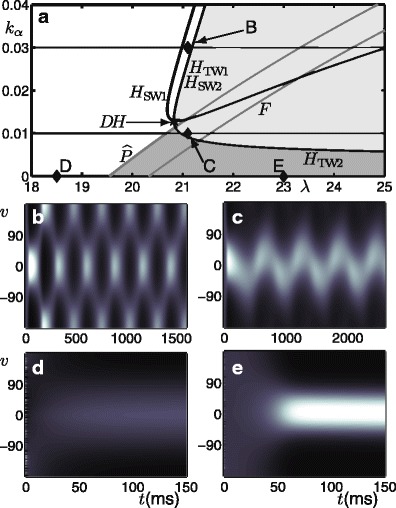
Bifurcation diagram for the simple input case with *k*_*I*_ = 0.001. **a** Bifurcation curves plotted in the (*λ*, *k*_*α*_)-parameter plane demarcate regions with qualitatively different dynamics. The Hopf curves are *H*_SW1_, *H*_TW2_, along with the coinciding *H*_SW2_ and *H*_TW1_; a further Hopf in the light-grey region involves only unstable solutions and is not labelled; all the Hopf curves meet at a the double Hopf point *DH*. Two bifurcation curves resulting from the symmetry breaking of a pitchfork bifurcation are $\widehat {P}$ and *F*; see Fig. 12 in Appendix B and accompanying text. Panels **b**–**e** show the activity *p*(*v*, *t*) indicated by intensity for model simulations at parameter values from the corresponding points B–E in panel **a**

Figure 3 shows a new bifurcation diagram after the introduction of the simple input *I*_1D_shown in Fig. 1c with input gain *k*_*I*_ = 0.001. We are interested to see how the solutions identified in the previous section change and how their organisation in parameter space has been modified. The most notable result is that much of the structure from the no-input case has been preserved, albeit with subtle changes that are now discussed. In the white region (to the left of *H*_SW1_ and $\widehat {P}$) there is now a low-activity response that is weakly tuned to the input centred at *v* = 0; see panel d. In the dark-grey region there is still a steady-state, tuned response, but now centred on the stimulus at *v* = 0. In the light-grey region the stable behaviour is still predominantly a travelling wave solution resembling those shown in Fig. 2b and c but with a slight modulation as the wave passes over the stimulus; the modulated solution will be shown later. Here we highlight a qualitatively different type of travelling wave solution that can be found close to the Hopf curve *H*_TW2_, whereby the wave has been *pinned* to the stimulated direction, as a so-called *slosher* state (Folias 2011); see panel c. Furthermore, an elongated region in parameter space has opened between *H*_SW1_and the coinciding curves *H*_sw2_and *H*_TW1_, in which the stable behaviour is a standing wave, with one of its peaks aligned to the stimulus at *v* = 0. In the parameter regime studied here, a change in stability of the standing wave solution occurs with the introduction of an input, however, we note that it has been proved in Curtu and Ermentrout (2004) that stable standing wave solutions can also exist without input in different parameter regions. In order to describe in further detail the changes to bifurcation structure that occur when the stimulus is introduced we also consider several one-parameter slices in *λ* (indicated by horizontal lines) at fixed values of *k*_*α*_taken from the diagrams already shown in Figs. 2a and 3a. The one-parameter bifurcation diagrams corresponding to these slices are in Appendix B where we give a more detailed account of way in which symmetries are broken when an input is introduced.

The bifurcation analysis with the same input studied throughout this section, as shown in Fig. 1c, continues in the next section. We present the case *k*_*I*_ = 0.01 but in the context of a motion stimulus.

## Competition model applied to the study of multistable motion

### Model of direction selectivity in MT

We extend the general study presented in Section 3 and demonstrate how the same model can be used to study a specific neuro-biological phenomenon for which perceptual shifts are observed. We now associate the model’s periodic feature space *v* ∈ [−*π*, *π*) with motion direction. We assume that the model’s activity in terms of time-evolving of firing rates *p*(*v*, *t*) are responses of direction-selective neurons in the middle temporal (MT) visual area. Indeed, MT is characterised by direction-selective neurons that are organised in a columnar fashion (Diogo et al. 2003). Here we only consider a feature space of motion direction and, thus, we assume the model responses to be averaged across physical (cortical) space. The chosen connectivity function (4) shown in Fig. 1b represents mutual inhibition between sub-populations of neurons associated with competing directions; this type of connectivity naturally gives rise to winner-takes-all responses tuned to one specific direction. Indeed, there is evidence that competing percepts have mutually inhibitory representations in MT (Logothetis et al. 1989; Leopold and Logothetis 1996). We use the models tuned response to dynamically simulate the mechanisms driving perception; cortical responses of MT have been linked specifically to the perception of motion (e.g. Britten (2003) and Serences and Boynton (2007)). We assume that over time any particular tuned response will slowly be inhibited as represented by the linear spike-frequency adaptation mechanism in the model. Furthermore, we assume there to be a fixed-amplitude stochastic fluctuation in the membrane potential that is modelled by additive noise (note that the noise is only introduced for the simulations presented in Section 5). We use as a model input pre-processed direction signals in the form expected from V1 (Britten 2003; Born and Bradley 2005). In Section 4.3 the model’s response properties in terms of its contrast dependence and direction tuning properties will be matched to what is known about the direction selective behaviour of MT neurons from physiological studies (Albright 1984; Sclar et al. 1990; Diogo et al. 2003).

### Definition of motion stimuli

**Fig. 4:**
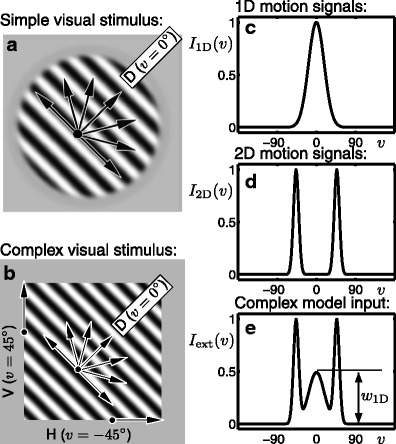
Simple and complex motion stimuli. **a** A drifting luminance grating viewed through a circular soft aperture; the diagonal direction of motion D is consistently perceived. **b** A drifting luminance grating viewed through a square aperture; the dominant percepts are vertical V, horizontal H and diagonal D. **c** Representation of the 1D motion signals in direction space; the simple motion stimulus **a** is equated with *I*_1D_. **d** Representation of the 2D motion signals in direction space. **e** Summation of the 1D and 2D motion signals with a weighting *w*_1D_ = 0.5; the complex motion stimulus **b** is equated with *I*_ext_

We introduce two classical psychophysics stimuli where a luminance grating drifting diagonally (up and to the right in the example shown) is viewed through an aperture see Figs. 4a and b. In the first case, with a circular aperture, the grating is consistently perceived as moving in the diagonal direction D (*v* = 0°). In the second case, the aperture is rectangular and tilted relative to the grating orientation. The classical barberpole illusion (Hildreth 1983, Chapter 4) comes about as a result of the aperture problem (Wallach 1935; Wuerger et al. 1996): a diagonally drifting grating viewed through an elongated rectangular aperture is perceived as drifting in the direction of the long edge of the aperture. With a square aperture, the stimulus is multistable for short presentations on the order of 2−3s, where the dominant percepts are vertical V (*v* = 45°), horizontal H (*v* = −45°) and D (*v* = 0°) (Castet et al. 1999; Fisher and Zanker 2001). We denote this stimulus the multistable barberpole and it has been the subject of complementary psychophysics experiments (Meso et al. 2012b) from which some results will be presented in Section 5.2.

It was shown in Barthélemy et al. (2008) that the motion signals from 1D cues stimulate a broad range of directions when compared with 2D cues that stimulate a more localised range of directions; see arrows in Figs. 4a and b. Based on these properties, it is proposed that the multistability for the square-aperture stimulus is primarily generated by competition between ambiguous 1D motion direction signals along grating contours on the interior of the aperture and more directionally specific 2D signals at the terminators along the aperture edges. We represent the 1D cues by a Gaussian bump $I_{\textrm {1D}}(v)=\exp (-v^{2}/2\sigma _{\textrm {1D}}^{2})$ with *σ*_1D_ = 18° centred at *v* = 0° as shown in Fig. 4c (which is the same as Fig. 1c); on its own we call this a simple input that represents a drifting grating either filling the visual field (without aperture) or with an aperture that has no net effect on perceived direction such as the circular one shown in Fig. 4a. We represent the 2D cues by two Gaussian bumps $I_{\textrm {2D}}(v)=\exp (-v^{2}/2\sigma _{\textrm {2D}}^{2})$ centred at *v* = 45° and *v* = − 45° with width *σ*_2D_ = 6° as shown in Fig. 4d. Note that the functions *I*_1D_ and *I*_2D_ are normalised such that their maxima are 1 (not their areas). Figure 4(e) shows the complex input *I*_ext_represented as a summation of 1D and 2D motion signals with maximum normalised to 1 and a smaller weighting *w*_1D_ ∈ [0, 1] given to 1D cues:

7 $$ I_{\text{ext}}(v)=w_{\textrm{1D}}I_{\textrm{1D}}(v)+I_{\textrm{2D}}(v-45)+I_{\textrm{2D}}(v+45). $$

The weighting *w*_1D_translates the fact that in motion integration experiments 2D cues play a more significant role that 1D cues in driving perceived direction of motion (Masson et al. 2000; Barthélemy et al. 2010). Here we represent this weighting in a simple linear relationship, but in future studies it may be relevant to consider the contrast response functions for 1D and 2D cues separately (Barthélemy et al. 2008).

### Simple input case with *k*_*I*_ = 0.01: parameter tuning for motion study and contrast dependence

**Fig. 5:**
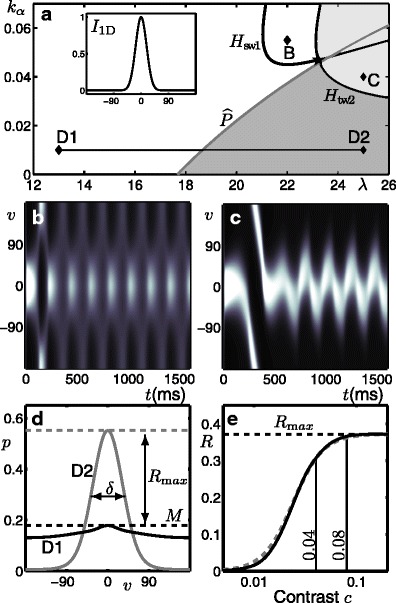
Bifurcation study and contrast response for simple input *I*_1D_ (see inset of **a**) with input gain *k*_*I*_ = 0.01. **a** Two-parameter bifurcation diagram in terms of sigmoid slope *λ* and adaptation strength *k*_*α*_ shows qualitatively the same organisation of bifurcation curves as Fig. 3a. **b**, **c** Time-traces of the activity *p*(*v*, *t*) indicated by intensity as computed at the corresponding points B and C labelled in **a**. **d** Steady-state responses in terms of the activity *p*(*v*) at the corresponding points D1 and D2 labelled in **a**. **e** Contrast response in terms of normalised peak activity *R* for simple input (*solid curve*) fitted to a Naka-Rushton function (*dashed curve*) as described in Appendix C. The line between D1 and D2 in **a** is the operating range of the model. The response D1 shown in panel **d** corresponds to *c* = 0 and the response D2 in panel **d** corresponds to *c* = 1

Figure 5a shows the two-parameter bifurcation diagram in *λ* and *k*_*α*_ for the same simple 1D input illustrated in Fig. 3a, but with the input gain increased by a factor of 10 to *k*_*I*_ = 0.01. The diagram shows the same organisation of bifurcation curves, but the oscillatory regions have shifted significantly towards the top-right corner. Once again, in the white region containing the point D1 there is a steady, low-activity, weakly tuned response (see lower curve in panel d). In the dark grey region containing the point D2 there is a steady, high-activity response with tuning width *δ* (see upper curve in panel d). We define *δ* as the width at half-height of the tuned response. Again, the boundary between these regions is demarcated by $\widehat {P}$ in Fig. 3a. In the region containing the point B there is still a standing-wave-type solution, but it is modified by the input such that there are breather-type oscillations (Folias 2011) between a tuned and an untuned state over time; see Fig. 5b. In this context there is no physiological interpretation for this solution, and so we will ensure that the model is operated in a parameter region where it cannot be observed. In the region containing the point C there is still a periodic response with small-amplitude oscillations in *v* of a tuned response about *v* = 0; see Fig. 5c. This is a travelling-wave-type slosher solution as described in Section 3.4 that is pinned by the input at *v* = 0°; note the transient that makes one full excursion before being pinned. Closer to the bifurcation curve the solutions are immediately pinned and further away a phase slip can be encountered as shown in Fig. 13g–i in Appendix B. For a simple (unambiguous) input we again operate the model away from this oscillatory region of parameter space but find that when the complex input is introduced it is the slosher-type solutions that produce the desired switching behaviour.

In order to produce results and predictions that can be related directly to the experiments, where contrast was one of the main parameters investigated, contrast should also be represented as a parameter in the model. In Curtu et al. (2008) the input gain *k*_*I*_ is assumed to depend on contrast and for some critical value of *k*_*I*_switching behaviour is observed. Here, we choose another option, arguing that motion signals arriving in MT, primarily from V1, are normalised by shifts in the sigmoidal nonlinearity (see Carandini and Heeger (2011) for a review) and therefore, the input gain *k*_*I*_ in Eq. (1) should remain fixed with respect to contrast. Indeed, by making the slope parameter *λ* depend on the contrast *c* ∈ [0, 1], we are also able to reproduce the observed switching behaviour with increasing contrast.

We now fix *k*_*α*_ = 0.01 such that we operate the model away from the oscillatory regions shown in Fig. 5 and describe how the model can be reparametrised in terms of contrast *c*. For some steady state $\bar {p}$, we define firing rate response $R=\max \{\bar {p}\}-M$ as the peak firing rate response above some baseline value *M*; $\max \{\bar {p}\}$ is shown as a dashed line for solutions D1 and D2 in Fig. 5d and we set $M=\max \{\bar {p}_{\textrm D1}\}$. As discussed in more detail in the Appendix C, the solution D1 at *λ* = 13 is consistent with an MT response to a very low contrast input (*c* < 0.01), whereas the solutions D2 at *λ* = 25 is consistent with a high contrast input (*c* > 0.2). By making *λ* a specific function of *c* we are able to match the model’s contrast response to known behaviour for MT neurons. As shown in Fig. 5e, we match the model’s response to an appropriately parametrised Naka-Rushton function, which was used to fit contrast response data across several stages of the visual pathway including MT in (Sclar et al. 1990); again, refer to Appendix C for further details. The operating range for the model is indicated by a horizontal line at *k*_*α*_ = 0.01 for *λ* ∈ [13, 25] in Fig. 5a. In Appendix C we also show that the tuning widths *δ* of the model responses are in agreement with the literature (Albright 1984; Diogo et al. 2003).

### Complex input with *k*_*I*_ = 0.01

**Fig. 6:**
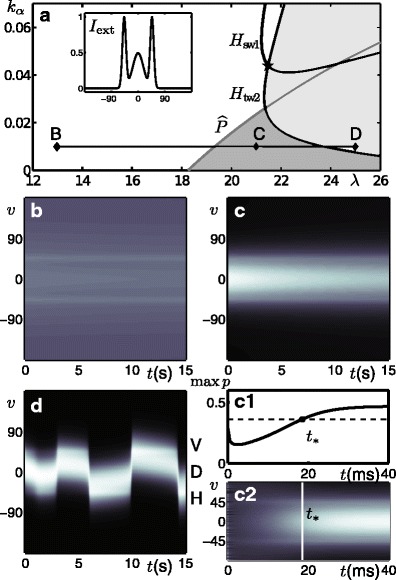
Bifurcation study for complex input *I*_ext_ (see inset of **a**) with input gain *k*_*I*_ = 0.01. **a** Two-parameter bifurcation diagram in terms of sigmoid slope *λ* and adaptation strength *k*_*α*_ shows qualitatively the same organisation of bifurcation curves as Figs. 3a and 5a. Line between points labelled B and D in **a** shows the operating region of the model as defined in Fig. 5a. **b**–**d** 15s time-traces of the activity *p*(*v*, *t*) indicated by intensity as computed at the corresponding points B, C and D labelled in **a**. Panel c1 shows the maximum of the activity and panel **c2** shows detail from **c** for the first 40ms of the simulation. The first direction tuned response coincides with the max of the activity crossing through a threshold value of $\max \{p\}=M+\frac {R_{\text {max}}}{2}$ as indicated by a horizontal dashed line in **c1**

**Fig. 7:**
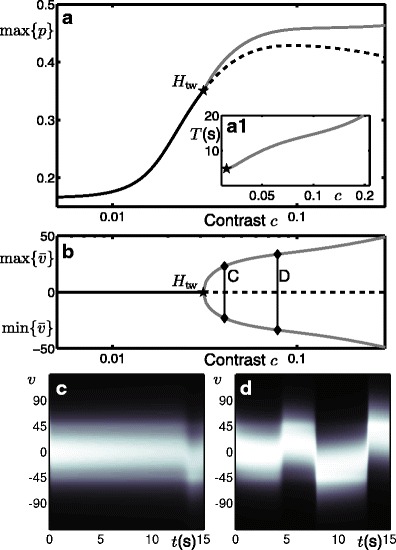
Bifurcation diagram with complex input for model parametrised in terms of contrast. **a**, **b** One-parameter bifurcation diagrams show the same data plotted in terms of the maximum response and average direction, respectively; the steady-state response tuned to D is solid black when stable and dashed black when unstable. Stable branch of oscillations between H and V is grey. **a1** The period on the oscillatory branch. **c, d** Time-traces of the activity *p*(*v*, *t*) indicated by intensity as computed at the corresponding points C and D labelled in **b**.

This section will focus on the bifurcation results for a complex input as shown in Figs. 6 and 7, but first we discuss the choice of timescale parameters. Up until now the results presented have been carried out with the main population time constant and adaptation timescale fixed at *τ*_*p*_ = 1ms and *τ*_*α*_ = 100ms, respectively. In the following sections the cortical time scale remains fixed at *τ*_*p*_ = 1ms and the adaptation timescale is tuned to a value of *τ*_*α*_ = 16.5s in order to match average switching rates in the experimental data presented in Section 5. Concerning the population time constant *τ*_*p*_, minimal neuronal latencies in response to flashed or moving high contrast stimuli are 40−45ms in macaque visual area MT, see Kawano et al. (1994) and Raiguel et al. (1999). From our simulations, if we look at the typical early dynamics as shown in Fig. 6c1 and c2, the transition to the first direction tuned response occurs at *t*_∗_ ≈ 20ms. The latency of this response depends both on *τ*_*p*_ and on the lateral spread of activity through the connectivity kernel, thus, the time constant we use is an appropriate order of magnitude. Concerning the adaptation timescale *τ*_*α*_, slow adaptation of firing rates in the visual cortex occur on timescales that range from 1−10s for excitatory neurons and 2−19s for inhibitory neurons (Sanchez-Vives et al. 2000; Descalzo et al. 2005). Yang and Lisberger (2009) have shown that a 10s presentation of a moving stimulus reduces neuronal response strength in macaque MT and that this has an effect on pursuit eye movements. Such a time course is consistent with human psychophysics data on the relationship between adaptation duration and strength in visual motion processing, see (Mather et al. 1998). The chosen adaptation timescale is of an appropriate order of magnitude with respect to visual motion adaptation in cortical areas as well as for visual perception.

The change in the value of *τ*_*α*_ from 100ms to 16.5s does not qualitatively affect the bifurcation diagrams shown in earlier sections, but in order for these results to be relevant it is desirable that we work with a small-amplitude additive noise as governed by *k*_*X*_. The single source of noise in the model evolves with its timescale *τ*_*X*_ set equal to *τ*_*α*_. This choice was found to have a pronounced affect on the switching dynamics without the need for large values of *k*_*X*_. Note that the time units displayed in figures up to this point are milliseconds, but will be seconds in the remainder of the paper.

Figure 6 shows the bifurcation diagram for a complex input and the different types of behaviour that are observed in the operating range of the model as defined in the previous section. In the presence of the complex input, we see the same four regions found for the simple input; compare Figs. 5a and 6a. The top-right-most region of the (*λ*, *k*_*α*_)-plane in which oscillations about *v* = 0° are observed has grown significantly. As for the simple input, there is a weakly tuned steady-state response in the region containing the point B and there is a tuned steady-state response in the region containing the point C; see panels b and c. However, the region containing the point D now shows an altered oscillatory behaviour. We see a model response that is initially centred at the direction D but after 2–3s shifts to H and proceeds to make regular switches between H and V, see panel d. The model’s separation of timescales is now seen more clearly; the model spends prolonged periods at H or V during which the adaptation builds up and eventually induces a switch to the opposite state; with *τ*_*α*_ = 16.5s switches occur every ≈ 3s, but the transition itself takes only ≈ 50ms. We note that the time between switches is shorter than the timescale of adaptation, which implies that the switches occur whilst the adaptation is dynamic, i.e. still rising (falling) for the selected (suppressed) direction. Due to the dynamics being deterministic in the absence of noise ( *k*_*X*_ = 0), the switches occur at regular intervals.

As described in the previous section we fix the operating region of the model with *k*_*α*_ = 0.01 and for *λ* ∈ [13, 25] as indicated by the horizontal line in Fig. 6a. As *λ* is increased from *λ* = 13 at b to *λ* = 25 at d (equivalently contrast increases from *c* = 0 to *c* > 0.2) there are transitions from a weakly tuned response at point B to a tuned response at point C to an oscillatory response at point D. For *c* > 0.2 the model response saturates as shown in Fig. 5e. We now incorporate another known aspect of contrast dependence in motion processing by varying the relative weighting between 1D and 2D cues in the input. The psychophysics experiments presented in Lorenceau and Shiffrar (1992) and Lorenceau et al. (1993) show that 1D cues (contour signals) play an important role in motion perception at low contrast that diminishes with increasing contrast. As contrast increases the 2D cues (terminator signals) play a more significant role. Based on these studies we propose that for the complex model input (7) the relative weighting of 1D cues should decrease linearly with contrast

8 $$ w_{\textrm{1D}}=W_{0}-W_{1}c, $$

where *W*_0_ = 0.5 and *W*_1_ = 1.1. These specific values were chosen in order to match the experiments; see further comments in Section 5.3.

Figure 7 shows a one-parameter bifurcation diagram for the model working in the operating regime shown in Figs. 5a and 6a but now reparametrised in terms of contrast *c* as described above. At low contrast there is a stable steady-state response tuned to the direction D. The peak response max{*p*} increases with contrast. This steady-state response loses stability at a travelling-wave Hopf instability *H*_tw_beyond which there is a stable oscillatory branch. The dependence of *w*_1D_ on the contrast affects the solutions in the two following ways. Firstly, the unstable branch associated with the D direction decreases in max{*p*} at large contrasts; see dashed curve in panel a. Secondly, the period and amplitude of the oscillations in $\bar {v}$ does not saturate but continues to increase with contrast as shown in the inset a1 and panel b. We also note that close to the bifurcation point *H*_tw_ there are long transients before the onset of oscillations, see panel c, and that further from the bifurcation point the onset of oscillations is faster, see panel d.

## Comparison of the model with experimental results

### Experimental results

**Fig. 8:**
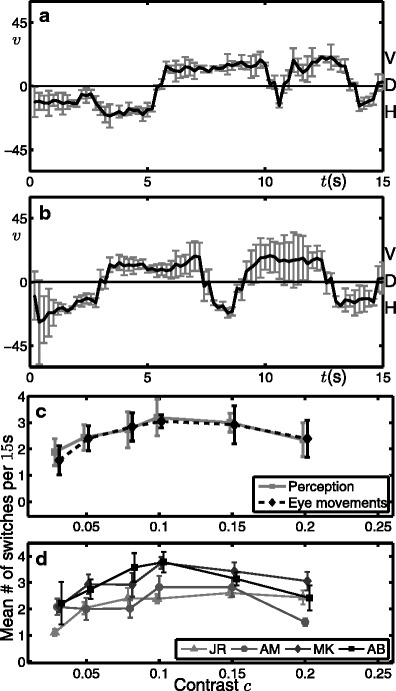
Summary of results from psychophysics experiments for the complex stimulus shown in Fig. 4b. **a, b** Time traces of average direction from eye-movements during two individual stimulus presentations at *c* = 0.08. Error bars show the standard deviation of the computed direction of smooth components over 200 samples; the re-sampled value at 5Hz is the mean. **c** Relation between contrast and mean switching rate in terms of *perception* (reported by subjects) and as computed from *eye-movement* traces; grouped data is averaged across the four subjects with standard error shown. **d** Switching-rate data (from perception) separated out by subject with standard error for each subject shown

Figure 8 shows a summary of experimental data obtained in psychophysics experiments with 15s presentations of the complex stimulus shown in Fig. 4b (Meso et al. 2012b). Four healthy volunteers who provided their informed consent were participants, of whom two were naive to the hypothesis being tested. All experiments were carried out with, and following CNRS ethical approval. An SR Eyelink 1000 video recorder was used for the eye movement recordings and psychophysics stimuli were presented on a CRT monitor through a Mac computer using Psychtoolbox version 3.0 running stimulus generation routines written in Matlab. Positions of the right eye were recorded and continuous smooth trajectories estimated after removing blinks, saccades (very fast abrupt movements) and applying a temporal low pass filter. The presented stimuli covered 10 degrees of visual angle (the size of the side of the square in Fig. 4b) and were presented at a distance of 57cm from the monitor. Each task was done over 8 blocks of up 15 minutes over which 36 trials spanning a range of six contrasts were randomly presented each time. In this paradigm, recorded forced choice decisions indicating shifts in perceived direction through the three directions H, D and V and the estimated eye directions were found to be coupled and both indicative of perceived direction. Further details of these experiments can be found in our previous presentation (Meso et al. 2012b) and a full description will appear in the experimental counterpart of this manuscript.

The temporal resolution of the eye traces is much higher than that of the reported transitions and allows for a relatively continuous representation of eye movement direction that can be compared with model simulations. Figure 8a and b show, for two different subjects, time traces of the time-integrated directional average of eye-movements from a single experimental trial at *c* = 0.08. Switches in perception can be computed from these trajectories by imposing thresholds for the different percepts. Both trials show that the directions H and V are held for extended durations and regular switches occur between these two states. The switches involve sharp transitions through the diagonal direction D. The diagonal direction can be held for extended durations immediately after presentation onset. However, we note that the eye-movement direction during the first 1s of presentation has a more limited history in its temporal filtering. Short presentations of the same stimulus were investigated in a related set of experiments (Meso et al. 2012a) and modelling work (Rankin et al. 2013).

Figure 8 also shows the relationship between the averaged rate of switches between H and V over a range of contrast values *c* ∈ {0.03, 0.05, 0.08, 0.1, 0.15, 0.2}; in panel c the data is averaged across the four subjects and in panel d it is separated out by subject. The lowest contrast shown *c* = 0.03 corresponds to the smallest contrast value for which subjects were able to reliably report a direction of motion for the stimulus. For the grouped data, at low contrast ( *c* < 0.1) the rate of switching increases with contrast with the rate being maximal at approximately *c* ≈ 0.1. Beyond the peak, for contrasts *c* > 0.1, the rate of switching decreases with contrast. For the data separated by subject shown in panel d, the subjects MK and AB have a peak rate around 3.5 switches per 15s presentation and the peak occurs at *c* ≈ 0.1. For subjects JR and AM the peak rate is lower at around 2.5 switches per 15s presentation and there is a less prominent peak occurring at a higher contrast value *c* > 0.1. However, the common pattern reveals two qualitatively different regimes with respect to changing contrast. A low contrast regime for which the switching rate increases with contrast and a high contrast regime for which the switching rate decreases with contrast.

### Model simulations with noise (*k*_*X*_ = 0.0025)

We now study the dynamics of the model in the presence of additive noise in the main neural field equation. Recall that the stochastic process in the model is operating on the same slow timescale *τ*_*α*_as the adaptation and that the strength of the noise is *k*_*X*_ = 0.0025. Two cases will be studied, first the low contrast case at *c* = 0.04, close to the contrast threshold on the steep part of the model’s contrast response; see Fig. 5e. Second, the high contrast case at *c* = 0.08, which is above the contrast threshold on the saturated part of the contrast response function. In the first case, noise is introduced in a parameter regime where the model is close to bifurcation and oscillations only occur after a long transient, see Fig. 7c. When operating in a nearby parameter regime close to bifurcation the noise causes random deviations away from the direction D and can drive the model into an oscillatory state more quickly. In the second case, noise is introduced in a parameter regime where the model produces an oscillatory response with a short transient behaviour, see Fig. 7d. In this regime the noise perturbs the regular oscillations either shortening or prolonging the time spent close to H and V.

**Fig. 9:**
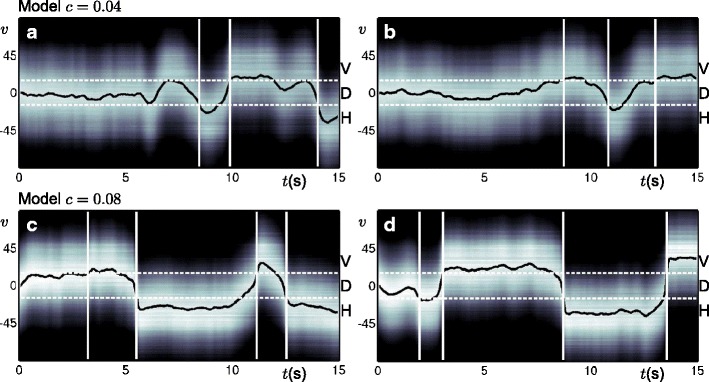
Time traces from individual model simulations where intensity shows the population activity across direction space (vertical axis). The solid black line is the average of this activity (average direction $\bar {v}$) and the dashed lines indicate perception thresholds (*PT*) for detection of switches between the directions H and V; switches are indicated by vertical white lines. First and second rows shows examples from the low and high contrast cases, respectively

Figure 9a–d shows 15s time traces of the population activity *p* for the cases *c* = 0.04 (first row) and *c* = 0.08 (second row). Note that each individual model simulation is quite different due to the noise, but we have selected representative examples that allow us to highlight key features in the model responses and compare the different contrast cases. In processing this simulated data we observe that the activity is initially centred around the direction D. After some transient period switching occurs primarily between H and V. In order to detect switches between the directions H and V a so-called perceptual threshold (*PT*) has been set at *v* = ± 10°. The first switch from D to either H or V is detected the when the corresponding threshold is crossed for first time. Subsequent switches are only detected the next time the opposite threshold is crossed. Note that although other algorithms could be employed to detect these switches, we found that these do not have a great effect on the presented results.

Across all the examples shown in Fig. 9, the average direction $\bar {v}$ oscillates in a random fashion and as time progresses the amplitude of these oscillations grows in *v*. For the case *c* = 0.04 there is a long transient and the first switch occurs for approximately *t* ∈ [5s, 10s]. For the case *c* = 0.08 the overall amplitude of the oscillations is larger and the first switch occurs for *t* < 3s. Note also that the level of activity shown as an intensity in Fig. 9 is higher in the *c* = 0.08 case. An important difference between the two contrast cases is that in the low contrast case, the transitions between H and V occur gradually when compared with the abrupt transitions in the high contrast case. This suggests that at low contrast the direction D could be seen during the transitions, where as in the high contrast case the switches occur directly from H to V.

With respect to the experimental data, the model consistently reproduces the characteristic behaviour of regular switches between the H and V. Furthermore, the sharp transitions through the diagonal direction D are also captured well by the model. Compare the second row of Fig. 9 with the two examples shown in Fig. 8a and b.

### Dependence of switching rate on contrast

**Fig. 10:**
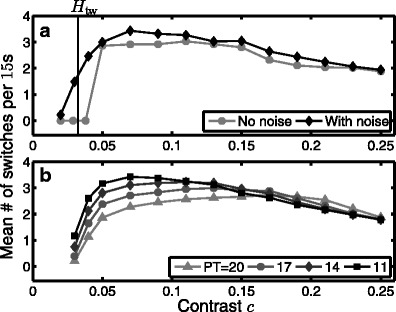
Mean switching rates computed with the model and recorded from psychophysics experiments. **a** Switching rates computed with the model without noise and with noise. **b** Switching rate curves computed with the model for a range of *PT* values

Figure 10 shows the relationship between contrast and switching rate as computed with the model where the rate is expressed as the mean number of switches per 15s simulation. Panel a shows the relationship without noise ( *k*_*X*_ = 0) and with noise ( *k*_*X*_ = 0.0025). We show the average switching rate at discrete contrasts *c* ∈ [0.02, 0.25] and at each contrast value we plot the switching rate averaged across 500 model simulations.

The deterministic (no noise) case can be explained in terms of the bifurcation diagram shown in Fig. 7. At low contrast, no switching behaviour is observed as the model can only produce a steady-state response weakly tuned to the direction D. With increasing contrast, the onset of switching is abrupt, occurring just above *c* = 0.04 after the bifurcation *H*_tw_ at *c* ≈ 0.03. Switching does not begin immediately at the bifurcation point, due to long transients for values of *c* nearby, see Fig. 7c. The switching rate remains constant at around 3 switches per 15s interval, and starts to drop off for contrasts *c* > 0.12. The reduction in switching rate for larger contrasts is due to the increasing period of the oscillations as shown in Fig. 7a1.

The introduction of noise with fixed intensity across all contrasts leads to an increase in average switching rate, see Fig. 10a. Here we only consider the average rate, but the distribution of the switching times will be discussed in Section 5.4. At larger contrasts, far from the bifurcation *H*_tw_, the switches are primarily governed by an underlying adaptation driven oscillation and the increase in switching rate is minimal. At lower contrasts, the shortest period of the oscillations is predicted to be immediately after the bifurcation; see Fig. 7a1. However, close to bifurcation, the theory also predicts that a long transient behaviour will be observed before the onset of these fast switches. The transient behaviour is important here, due to the fact that we consider short 15s simulations. At very low contrasts, before *H*_tw_, only the noise can drive a deviation from the diagonal direction leading to a switch. However, just after the bifurcation *H*_tw_, the main contribution to the increased switching rate is noise shortening the transient period before the onset of adaptation-driven switching. Therefore, the bifurcation analysis provides an explanation for the fact that the peak in switching rate occurs shortly after the bifurcation where transients are curtailed by the noise and the period is shortest.

The model results with noise are able to accurately capture the two contrast regimes from the experimental data: an increase in switching rate at low contrasts and subsequent decrease in switching rate at higher contrasts, compare Fig. 10a black curve with Fig. 8a. The values of *W*_0_, *W*_1_ and *τ*_*α*_ were chosen in order to fit the experimental data, however, the two contrast regimes are robustly produced by the model independent of the specific values chosen. In Fig. 10(b) we show how, in the model, the relationship between switching rate and contrast changes with respect to *PT*. When *PT* is low the peak switching rate is highest and occurs at a low contrast value. As *PT* is increased, the peak rate decreases and also occurs at a higher contrast value; the relationship also appears to flatten out for larger *PT*. Figure 8b shows the reported switching rate curves from the experiments, separated out by individual subject. The data shows a range of peak switching rate between the subjects. For the two subjects with the highest switching rate (MK,AB), the prominent peak occurs at *c* ≈ 0.1. For the other two subjects (JR,AM), the peak rate is lower, the response is flatter and the peak rate occurs at a larger value of *c*. We conclude that differences in perceptual threshold between subjects can account for inter-subject differences.

### Distribution of switching times

In the previous section, we showed example model outputs for which switches between the directions H and V are detected. We found that the times between these switches vary and that, particularly in the low contrast case, the early transient behaviour can be very different from one simulation to the next. In order to investigate the distribution of the switching times we ran 1, 500 model simulations each of 15s and formed a data set by extracting the times between consecutive switches from each simulation.

**Fig. 11:**
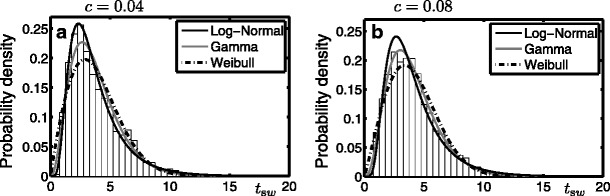
Distribution of perceptual switching times and test distributions. Histograms show the distribution of switching times as computed from model simulations with *PT* = 15° (see text for details). Candidate distributions are overlaid, where the shape and scale parameters have been chosen to best fit the model data. **a** Low contrast case in which the log-normal distribution provided a better fit. **b** High contrast case in which the gamma distribution provided a better fit

**Fig. 13:**
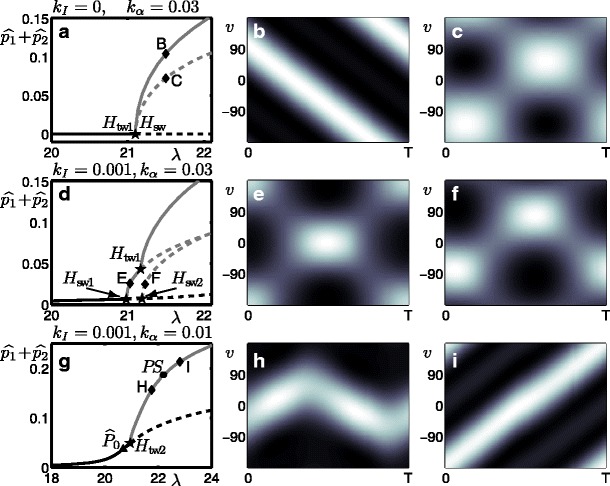
Changes to standing- and travelling-wave branches born in Hopf bifurcations with introduction of the stimulus. The first column shows one-parameter bifurcation diagrams in *λ* where black curves are steady-state branches and grey periodic branches; stable solution branches are solid and unstable branches are dashed. Hopf bifurcations to travelling waves are *H*_tw1_ and *H*_tw2_, and to standing waves are *H*_sw_, *H*_sw1_, and *H*_sw2_. Second and third columns show one period *T* of the solutions at corresponding points on solution branches from one-parameter diagrams

Figure 11 shows histograms of the computed switching times *t*_sw_. In the low contrast case approximately 1, 483 switches were recorded with mean time $\bar {t}_{\text {sw}}=3.73$s and SD = 2.89 (Coefficient of Variance COV = 0.56) and in the high contrast case 3, 154 switches were reported with mean time $\bar {t}_{\text {sw}}=4.07$s and SD = 2.08 (COV = 0.51). Although the mean of *t*_sw_ is smaller in the high contrast case, more switches are detected because there is a shorter transient period before switching begins; the average time to the first switch in the low contrast case is 7.47s (SD = 2.87) compared with an average time of 3.02s (SD = 1.56) in the high contrast case. The aim now is to determine from which distribution the model data could have arisen. We follow the method presented in (Shpiro et al. 2009) and compare the model data with a Weibull probability distribution function (pdf), a gamma pdf and a log-normal pdf each with parameters chosen using a standard maximum likelihood estimate. By inspection, it appears that the data in the low contrast case are well fitted by a log-normal distribution and that the data in the high contrast case are well fitted by a gamma distribution. In order to confirm this we perform a Kolmogorov-Smirnov goodness-of-fit test. In the low contrast case the log-normal distribution provides best fit ( *P* = 0.087) but the gamma and Weibull distributions can be rejected at the 5*%* significance level. In the high contrast case the gamma distribution provides best fit ( *P* = 0.635) and both the log-normal and Weibull distributions can be rejected.

Clearly, studying only the mean and standard deviation for the two different contrast cases does not reveal a significant difference. However, we do find a change in the underlying distributions governing the switching times, which is indicative of a change in the dominant mechanism driving the switching. Typically switching behaviour that is driven by adaptation over noise will have a lower peak that occurs later and a smaller spread with shorter tail as characterised by the gamma distribution (Shpiro et al. 2009). However, when noise plays a more significant role, the peak is higher, earlier and the tail longer as characterised by the log-normal distribution. We also highlight the fact that the first switch occurs much earlier in the high-contrast case, this prediction could easily be tested experimentally.

## Discussion

Spatially extended neural fields models with a linear implementation of spike-frequency adaptation have been studied both in ring models (Hansel and Sompolinsky 1998; Curtu and Ermentrout 2004; Kilpatrick and Ermentrout 2012; Ermentrout et al. 2012) and infinite spatial domains (Pinto and Ermentrout 2001; Ermentrout et al. 2012). A simpler version of the model presented in this article, without an input or noise, was studied in Curtu and Ermentrout (2004). The existence of parameter regions with homogeneous, stationary tuned, travelling-wave and standing-wave responses was shown. In the absence of an input, these various different solution types are known to exist close to a so-called Bogdanov-Takens (BT) point in parameter space, which acts as a parametric organising centre for different types of dynamics. In the presence of simple inputs it has been shown that new solution types can be produced such as breathers and pinned travelling-wave solutions (Hansel and Sompolinsky 1998; Ermentrout et al. 2012). In this article we presented an in-depth numerical study of the complex organisation of these various solution types in three-dimensional parameter space. We were able to show that much of the structure local to the BT point is preserved with the introduction of a small, simple input, albeit in a subtly modified form. We gave an account of the changes that occur in terms of the complicated series of bifurcations that delineate regions of parameter space exhibiting qualitatively different dynamics. It was found that close to a travelling-wave-type Hopf bifurcation, solutions are pinned to the input. Furthermore, it was shown that for a standing-wave-type Hopf bifurcation giving rise to unstable solutions with no input, a region in parameter space with stable standing waves solutions was opened up when an input was introduced. Although great progress has been made analytically in the study of this class of model for simple inputs where a single location in feature space is stimulated (Hansel and Sompolinsky 1998; Ermentrout et al. 2012), the question of more complex inputs with stimulation of multiple locations provides a challenge. The advantage of the numerical approach used here is that we can directly extend earlier results when a complex input is introduced. In a recent study, perceptual multistability has been investigated in a model with synaptic depression and a two-location stimulus in a continuous feature space (Kilpatrick 2012).

We subsequently investigated perceptual multistability for a stimulus that is multistable in terms of its perceived direction of motion and that has been the subject of recent psychophysical experiments, of which a summary was presented (see Meso et al. (2012b)). This specific application allows for the model’s continuous feature space to be exploited; it allows for a truly dynamic consideration of perceived direction, which unlike binocular rivalry or ambiguous shapes, is known to be neurally represented on a continuous scale. We study the multistable barber pole, which consists of a diagonally drifting grating viewed through a square aperture. The stimulus is known to be multistable between the diagonal grating direction D and the horizontal H and vertical V aperture-edge directions. The characteristic perceptual response is dominated by D immediately after onset followed by regular switches between horizontal H and vertical V directions. In the model the complex multistable barberpole can be represented by three-bumps in the feature space of motion direction based on experimental insights about the different ways in which 1D and 2D motion cues are processed by the visual system. The simple input is used to tune model parameters and introduce a contrast parameter such that its behaviour matches the known contrast response properties from (Sclar et al. 1990). It is found that for a fixed adaptation strength, we are able to select a range of the nonlinear slope parameter such that the model’s activity response can be matched to the qualitative and quantitative behaviour close to contrast threshold observed in physiological experiments. Once appropriately parametrised for the simple input, we find that for a complex input the model produces behaviour that is consistent with the characteristic perceptual responses described above.

We further investigated the relationship between contrast and the switching behaviour; in the experiments two different regimes were identified for the first time, at low contrast the switching rate increases with contrast and at higher contrasts the rate decreases with contrast. In the model we study a low contrast regime operating close to bifurcation and a high contrast regime above the contrast threshold. For both regimes we find common features in the switching behaviour produced by the model. Initially the percept D is dominant, but after some delay there is a shift to either H or V, typically within the first 1 − 8s (this behaviour is consistent with existing studies (Castet et al. 1999; Fisher and Zanker 2001; Rankin et al. 2013)), after which regular switching occurs between H and V. In the high contrast case this regular switching starts earlier, which we would expect as the 2D cues associated with the aperture edges and H/V directions should be stronger with increased contrast. We also find that typically the transitions between H and V are relatively smooth, passing gradually through the direction D in the low contrast case when compared with the sharper transitions in the high contrast case. Furthermore, by studying the dynamics either with or without noise, we find that at high contrasts the mean rate of switching is governed by the adaptation-driven oscillations. Although the noise produces random deviations in these switching times, the mean rate is unaffected. However, at low contrast, where the model is operating close to bifurcation the noise has a larger effect on the dynamics. At low contrast the increasing regime is associated with noise curtailing transient behaviour driving the model into an oscillatory regime. We further quantified the difference between the two contrast regimes by showing that the switching times are best fitted by a log-normal distribution in the low contrast case and by a gamma distribution in the high contrast case.

## Conclusions

In classical rivalry models competing states are modelled as discrete populations (Laing and Chow 2002; Shpiro et al. 2007; Moreno-Bote et al. 2007; Shpiro et al. 2009). The neural fields model at the core of this study has a continuous feature space, which allows multistability to be investigated in a motion integration problem where the different percepts are represented on a continuous scale. The minimal model incorporating spike frequency adaptation, additive noise and an input representing the multistable barberpole can capture characteristics of the switching observed in experiments: extended periods spent at the stimulated directions associated with different percepts and rapid switches between them. The bifurcation analysis allows for these dynamics to be related back to the travelling-wave-type solutions that are first pinned by a simple input and further modulated by the complex input in order to produce the desired behaviour. The bifurcation analysis predicts a change in the mechanisms driving switches between a low contrast and a high contrast regime characterised respectively by increasing and decreasing switching rates in the psychophysical experiments. The switches are driven primarily by noise at low contrast and adaptation at high contrast. The peak switching rate is predicted by the bifurcation analysis to occur just after bifurcation where the fastest adaptation-driven dynamics are reached after transients that are shortened by noise; in effect, when there is a balance between adaptation and noise.

The general approach applied to a neural field model in this paper — making use of bifurcation methods for tuning parameters such that the model operates close to bifurcation whilst simultaneously matching known response properties from physiological studies — will allow for much broader studies of multistable perception. In particular, extensions to models that consider physical space in conjunction with an abstracted feature space would allow for the particular spatial properties of multistable visual stimuli to be investigated. For example, more detailed neural fields models taking into account the spatial integration of motion stimuli such as (Tlapale et al. 2011) could be used to, within a single model architecture, investigate the underlying motion integration mechanisms that yield multistable perception across a broad range of stimuli, e.g., barberpoles, plaids, moving diamonds. Multistability has not been investigated in this kind of model to which the methods of numerical continuation and bifurcation analysis would be most applicable.

## Figures and Tables

**Fig. 12:**
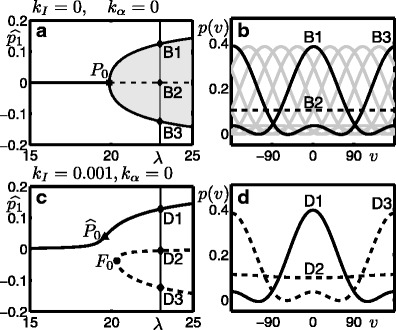
Symmetry breaking of the pitchfork with introduction of a stimulus. **a** and **c** show bifurcation diagrams in *λ* for the no-input and small input cases, respectively; stable states are solid curves and unstable states are dashed curves. **b** and **d** show the solution profiles in *v*-space at the labelled points for *λ* = 23; stable states are solid curves and unstable states are dashed curves. In panel **b** the solid black curves correspond to the solution B1, for which $\widehat {p}_{1}$ takes its largest value and the solution B3, for which $\widehat {p}_{1}$ takes its smallest value; several intermediate solutions are plotted as grey curves, see text

**Fig. 14:**
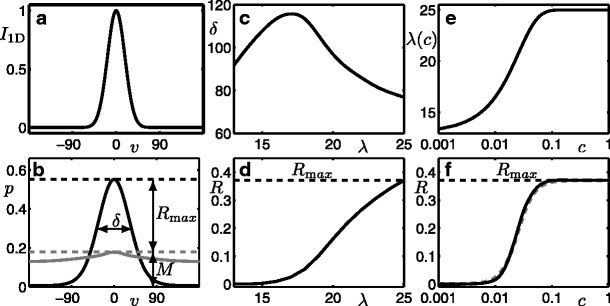
Model response for drifting grating stimulus and tuning of *λ*-dependence on *c*. **a** Stimulus profile *I*_1D_(*v*) for a visual-field-covering drifting grating. **b** Steady state responses at *λ* =13 (grey curve) and at *λ* =25 (black curve); dashed horizontal lines indicate the maximum activity for these steady states. The tuning bandwidth, or tuning width at half-height, is *δ* and the maximum level of activity above the spontaneous level (dashed grey line) is *R*. **c** Tuning width *δ* of response for *λ* ∈ [*λ*_min_, *λ*_max_]. **d** Normalised response *R* for *λ* ∈ [*λ*_min_, *λ*_max_]. **e** Variation of *λ* with respect to *c*. **f** Contrast response curve with *λ* varying as in panel **e** (*black*) and the Naka-Rushton function with parameters as described in the text (*dashed grey*)

## Appendix

### A The Ornstein-Uhlenbeck process

The noise *X*(*v*, *t*) that appears in Eq. (1) is a classical Ornstein-Uhlenbeck process, see e.g. (Ermentrout and Terman 2010), that is described by the following stochastic differential equation

9 $$ \tau_{\alpha} \mathrm{d} X(v,t)=-X(v,t)\mathrm{d} t+\sigma \mathrm{d} W(v,t), $$

where *W*(*v*, *t*) is a feature uncorrelated Wiener or Brownian process. Note that the timescale *τ*_*α*_ is the same as the one for the adaptation *α* in Eq. (2).

The solution to Eq. (9) is readily found to be

$$X(v,t)=e^{-t/\tau_{\alpha}}X_{0}(v)+\sigma \int_{0}^{t} e^{-\frac{t-s}{\tau_{\alpha}}} dW(v,s),\quad t \geq 0 $$

where *X*_0_(*v*) is the initial noise distribution, assumed to be independent of the Brownian. Its mean is therefore given by

$$\langle X(v,t) \rangle = \langle X_{0}(v) \rangle e^{-t/\tau_{\alpha}}, $$

and its variance by

$$\text{Var}(X(v,t))=\text{Var}(X_{0}(v)) e^{-2t/\tau_{\alpha}}+\frac{\tau_{\alpha} \sigma^{2}}{2}(1-e^{-2t/\tau_{\alpha}}) $$

It is seen that as soon as *t* becomes larger than the timescale *τ*_*α*_ the mean becomes very close to 0 (it is even exactly equal to 0 for all times if the mean of the initial value is equal to 0), and the variance becomes very close to $\frac {\tau _{\alpha } \sigma ^{2}}{2}$. By choosing *σ* ^2^ = 2 / *τ*_*α*_ we ensure that Var(*X*(*v*, *t*)) is close to 1 as soon as *t* becomes larger than the timescale *τ*_*α*_.

### B Explanatory one-parameter bifurcation diagrams

Figure 12 shows one-parameter bifurcation diagrams with zero adaptation gain *k*_*α*_ = 0, first with no input in panel a and with a small simple input ( *k*_*I*_ = 0.001) in panel c. In order to best represent the solution branches we plot them in terms of the even, first-order mode of the solutions $\widehat {p_{1}}$ (the cos(*v*)-component). Figure 12a shows that for small *λ* there is a single, stable solution branch with $\widehat {p_{1}}=0$; this corresponds to the flat (untuned) response shown in Fig. 2d. When *λ* is increased beyond the pitchfork *P*_0_, this flat state loses its stability and a ring of tuned responses are created. Figure 12b shows the profiles of the different solutions that exist at *λ* = 23. The dashed curve B2 is the unstable flat state, the tuned state B1 centred at *v* = 0 corresponds to when $\widehat {p_{1}}$ is largest and the tuned state B3 centred at *v* = ± 180 corresponds to when $\widehat {p_{1}}$ is smallest. Due to the presence of translational symmetry, intermediate states centred at any value of *v* also exist; discrete examples of these are shown as grey curves, but note these exist on a continuous ring filling in the direction space *v*. The easiest way to see the effect of introducing the stimulus and the fact that this breaks the translational symmetry is by studying the states that exist in the small input case also at *λ* = 23 shown in Fig. 12d. Now the only stable solution is the tuned response D1 centred at *v* = 0, there is a counterpart unstable solution centred at *v* = ± 180 and all of the intermediate states have been destroyed. In the bifurcation diagram Fig. 12c the pitchfork bifurcation has been destroyed and there remain two disconnected solution branches. On the unstable branch the unstable solutions D2 and D3 are connected at a fold point *F*_0_; this bifurcation is traced out as *F* in Fig. 3a. On the (upper) stable branch there is a smooth transition with increasing *λ* from a weakly- to a highly-tuned response centred at the stimulated direction. It is useful to detect where the increase in $\widehat {p_{1}}$ is steepest as this signifies the transition to a tuned response. We denote this point $\widehat {P}_{0}$ and as this is not strictly a bifurcation point we call it a pseudo pitchfork; it is still possible to trace out where this transition occurs in the (*λ*, *k*_*α*_)-plane and this is plotted as $\widehat {P}$ in Fig. 3a.

Figure 13 shows one-parameter bifurcation diagrams in *λ* for three different cases:

- No input cases with *k*_*α*_ = 0.03; see Fig. 13a–c; corresponds to the horizontal line through the point labelled B in Fig. 2a.
- First small, simple input case with *k*_*α*_ = 0.03; see Fig. 13d–f; corresponds to the horizontal line through the point labelled B in Fig. 3a.
- Second small, simple input case with *k*_*α*_ = 0.01; see Fig. 13g–i; corresponds to the horizontal line through the point labelled C slice in Fig. 3a.

In order to best represent the solution branches we plot them in terms of the maximum of the sum of the even and odd first-order mode of the solutions $\max \{\widehat {p_{1}}+\widehat {p_{2}}\}$ (the cos(*v*) and sin(*v*)-components). Figure 13a shows that two solution branches bifurcate simultaneously off the trivial branch at the twice-labelled point *H*_tw1_ *H*_sw_. In panel b we show one period of a stable travelling wave solution from the branch corresponding to *H*_tw1_; the wave can take either positive or negative (shown) direction in *v*. In panel c we show one period of an unstable standing wave solution from the branch corresponding to *H*_sw1_; the standing wave oscillates such that 180°-out-of-phase (in *v*) maxima form alternatively. The phase in *v* of the entire waveform is arbitrary and, as for the pitchfork bifurcation, any translation of the whole waveform in *v* is also a solution. With the introduction of a stimulus, as for the pitchfork bifurcation discussed earlier, the translational symmetry of the solutions is broken resulting in changes to the solution structure. The bifurcation point *H*_sw_ shown in Fig. 13a splits into two bifurcation points *H*_sw1_ and *H*_sw2_ in panel d and solution profiles on the respective bifurcating branches are shown in panels e and f. On the first branch, which is initially stable close to *H*_sw1_, one of the maxima is centred on the stimulated direction *v* = 0°. For the second unstable branch bifurcating from *H*_sw2_ the maxima are out of phase with the stimulated direction. The travelling wave branch is now a secondary bifurcation from the branch originating at *H*_sw1_ and the solutions now have a small modulation when the travelling wave passes over *v* = 0° (similar to the solution shown in panel i). In panel g, at a lower value of *k*_*α*_, the steady-state solution branch is initially weakly tuned and forms a highly-tuned solution after $\widehat {P}_{0}$. The tuned response becomes unstable at *H*_tw2_; close to this bifurcation point the solution is pinned by the stimulated direction, as was shown in Fig. 3c, and as *λ* is increased further the amplitude in *v* of these oscillations about the stimulated direction increases, see panel Fig. 13h. When the point *P* *S* is reached in panel h the oscillations become large enough such that there is a phase slip and beyond this point we obtain a standard travelling wave solution once more, see panel i. Note that the travelling wave is still modulated as it passes over the stimulated direction. We reiterate the qualitative difference between the branches forming at *H*_tw1_ and *H*_tw2_: for branches of travelling wave forming directly from an untuned or weakly tuned steady state as at *H*_tw1_ the solutions cannot be pinned to a stimulated direction, however, for branches of travelling wave forming directly from a tuned steady state as at *H*_tw2_ the solutions are pinned to a stimulated direction close to the bifurcation. Furthermore, we note that, with the introduction of a stimulus a region of stable standing wave solutions, in phase with the stimulated direction, are introduced between *H*_sw1_ and *H*_tw1_; see branch segment through the point E in Fig. 13d.

### C Tuning the model’s contrast response

Here we describe the way in which the model is reparametrised such that its response matches known physiological behaviour. In (Sclar et al. 1990) contrast response functions were computed across several stages of the visual pathway for a drifting sinusoidal grating. At cortical layers, individual cell recordings were made with the stimulus moving in the cell’s preferred direction. The Naka-Rushton function was used to fit contrast response data and the response *R* as a function of contrast *c* is given by

10 $$ R(c)=R_{\text{max}}\frac{c^{n}}{c^{n}+c^{n}_{50}}+M, $$

where *R*_max_ is the maximal response $c^{n}_{50}$ is the contrast at which the response reaches half its maximum value and *n* is the exponent that determines the steepness of the curve. For visual area MT in the macaque these parameters were estimated to take the following values: *R*_max_ = 36, $c^{n}_{50}=0.07$ and *n* = 3; the Naka-Rushton function is plotted for these values in Fig. 14f (dashed curve). Note that it is also necessary to adjust for the spontaneous firing rate *M*, which typically takes a value *M* ∈ [0.05, 0.3].

We wish to reproduce the known contrast response properties for a drifting grating stimulus represented by the input in Fig. 14a. For very low contrasts *c* < 0.01, the model should respond to a stimulus with a low level of activity that is weakly tuned or not tuned. Increasing *c* should result in a change in the model’s response that is consistent with crossing the contrast threshold:

1. an increasing level of activity consistent with the Naka-Rusthon function Eq. (10),
2. a response that is tuned to the model’s input with an appropriate tuning width (consistent with values reported in (Albright 1984; Diogo et al. 2003)).

We find that, for *k*_*α*_ = 0.01 and *λ* = 13, the model’s weakly-tuned, steady state response has an appropriate level activity that we take as the value for *M* = 0.18; see the lower curve in Fig. 14b. Furthermore, changing to *λ* = 25 we find that the model produces a tuned response that is consistent with a response above contrast threshold *c* > 0.2 with a maximum level of activity that agrees with the value of *R*_max_ = 0.52 − *M* = 0.36; see upper curve in Fig. 14b. We also find that varying *λ* in the range *λ* ∈ [13, 25] that the tuning widths at half-height *δ* fall in the range *δ* ∈ [80°, 115°] (see Fig. 14c), which is consistent with the tuning widths obtained by individually probing a population neurons in the studies (Albright 1984) (mean 90°, standard deviation SD = 29°) and (Diogo et al. 2003) (mean 104°, SD = 35°). Figure 14d shows the model’s response *R* plotted against *λ* ∈ [13, 25] and we see that initially, close to *λ*_min_ = 13, the response has an appropriate shape, but for larger *λ* the response does not saturate as required. It is therefore necessary to introduce a function *λ*(*c*) that saturates smoothly at the value *λ*_max_ = 25. We reparametrise the model so that *λ* depends on the contrast using a function that increases linearly from *λ* = *λ*_min_ and saturates for large *c* at *λ* = *λ*_max_. We effectively use the top half of a sigmoid function *S*(*x*) = 1 / (1 + exp( − *x*)). After fixing *λ*_min_ = 13 and *λ*_max_ = 25, it suffices to vary just the slope parameter *μ* in the following equation in order to find a good fit. The equation

11 $$\lambda(c)=\lambda_{\text{min}}+(\lambda_{\text{max}}-\lambda_{\text{min}})\left(S(\mu c)-\frac{1}{2}\right), $$

is plotted in Fig. 14e with *μ* = 60. By construction, we have set dependence of *λ* on *c* in order to obtain a function of the model response *R* in terms of *c* that matches a Naka-Rushton function appropriately parametrised for MT; see Fig. 14f (solid curve).
